# Diagnostic and Therapeutic Ambiguities in Diabetic Ketoacidosis With Overlapping Acute Pancreatitis and Hypertriglyceridemia

**DOI:** 10.7759/cureus.57508

**Published:** 2024-04-03

**Authors:** Michael Sabina, Amanda Rigdon, Joshua Tsai

**Affiliations:** 1 Internal Medicine, Lakeland Regional Health Medical Center, Lakeland, USA

**Keywords:** diabetic ketoacidosis, smv thrombosis, insulin, hypertriglyceridemia, pancreatitis, diabetes, critical care

## Abstract

This case report discusses the diagnostic challenges and management complexities in a patient presenting with symptoms of diabetic ketoacidosis (DKA) and severe pancreatitis, complicated by concurrent hypertriglyceridemia (HTG) and superior mesenteric vein (SMV) thrombosis. The presence of DKA in acute pancreatitis suggests very severe impact on the pancreas. Hence, it calls for screening with CT imaging for complications like hemorrhagic pancreatitis, necrotizing pancreatitis, or even thrombus. Despite typical reliance on clinical presentation and serum lipase for diagnosing pancreatitis, this case emphasizes the necessity of contrast-enhanced CT imaging in ambiguous cases to identify critical complications like thrombosis and necrotizing pancreatitis. Furthermore, the patient's management involved insulin therapy for DKA and HTG-induced acute pancreatitis, deferring plasmapheresis and anticoagulation due to the risk of hemorrhagic transformation in pancreatitis. This approach highlights the need for individualized treatment strategies, especially in complex presentations with overlapping pathologies. The case also explores the potential for insulin as a first-line treatment in HTG-induced pancreatitis over plasmapheresis, contributing to evolving guidelines.

## Introduction

Patients presenting with acute pancreatitis typically exhibit epigastric abdominal pain, and elevated serum lipase levels that are threefold times above baseline [1]. These clinical findings often suffice for a diagnosis of pancreatitis. However, this diagnostic approach may require reassessment in cases where the clinical presentation is obscured by overlapping pathologies such as diabetic ketoacidosis (DKA), which can mimic the abdominal pain associated with pancreatitis [2]. It is crucial to acknowledge that pancreatitis complications, including hemorrhagic and necrotizing forms, pseudocysts, and infection, significantly elevate mortality risk [3]. In instances of suspected chronic pancreatitis, the likelihood of complications such as splenic vein or superior mesenteric vein (SMV) thrombosis is increased [4]. These complications are typically detectable via contrast-enhanced computed tomography (CT) scans. While an overlapping condition such as DKA is present, which can mimic the abdominal pain presentation of pancreatitis, the decision to order a CT scan of the abdomen becomes necessary for proper diagnosis. Additionally, the management of severe HTG-induced acute pancreatitis typically involves insulin therapy or plasmapheresis, which makes the decision of which to initiate rather easy since insulin is the first-line treatment for DKA as well. Furthermore, the cornerstone of treating SMV thrombosis, as recommended by the American Gastrointestinal Association, is anticoagulation for a duration of 3-6 months [5]. This guideline, however, may require reconsideration in the context of a high risk for hemorrhagic pancreatitis. In this case report, we delve into our choices regarding diagnostic measures and management strategies. 

## Case presentation

We present a case of a 35-year-old male with no significant past medical history, who presented with intractable vomiting and severe abdominal pain that began 4 hours ago. He has also noted marked weight loss, excessive thirst, and frequent urination for approximately 11 months, occurring intermittently roughly every other week. Notably, he exhibited xanthelasma palpebrarum around his eyes, a condition present since his teenage years. He reports a family history of hypertriglyceridemia (HTG) and pancreatitis in his mother and sister.

Vitals on arrival were remarkable for a respiratory rate of 30, heart rate of 134, blood pressure of 151/94 mmHg, and oxygen saturation of 95% on room air. Laboratory findings revealed hyperglycemia with a blood sugar level of 582 mg/dL, an elevated anion gap of 26 mmol/L, bicarbonate level of 17 mmol/L, beta-hydroxybutyrate level of 13.6 mg/dL, and an increased lactic acid level of 6 mg/dL, pointing towards DKA. An A1C level of 12.2% further indicated new-onset, poorly controlled diabetes. An arterial blood gas (ABG) was performed and showed a pH of 7.36, and partial pressure of carbon dioxide (pCO2) of 36.1 mmHg, confirming our suspicion of metabolic acidosis with respiratory compensation, secondary to DKA.

The lipid panel revealed triglycerides exceeding 4425 mg/dL, with indeterminate low-density lipoprotein (LDL) due to hypertriglyceridemia. This raised concerns for possible HTG-induced pancreatitis. While the abdominal pain could have been attributed to DKA, a lipase level of 749 U/L required another diagnostic modality to rule out pancreatitis. Moreover, although low on our differential, we could not rule out acute mesenteric ischemia without imaging. We believed the lactic acidosis was secondary to hypoperfusion. There was only a subtle elevation in the white blood cell count of 11.1 x10^3/uL, no complaints of dark stools, blood-tinged stools, and a hemoglobin of 17.4 mg/dL. Initial chest x-ray on admission did not show signs of pneumatosis.

**Figure 1:**
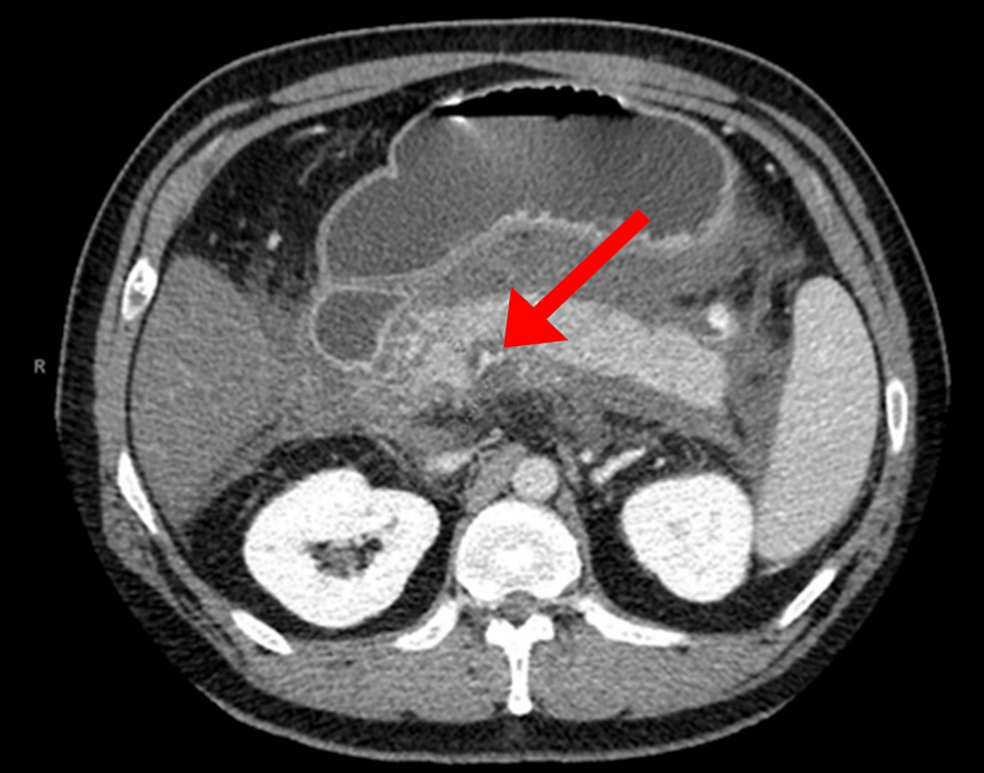
Computed Tomography Scan of Abdomen: Superior Mesenteric Vein Thrombosis (red arrow)

An abdominal CT scan with contrast was pursued and revealed severe pancreatitis with surrounding edema, along with splenic vein thrombosis and superior mesenteric vein thrombosis (Figure 1), suggesting chronic pancreatitis. 

**Figure 2:**
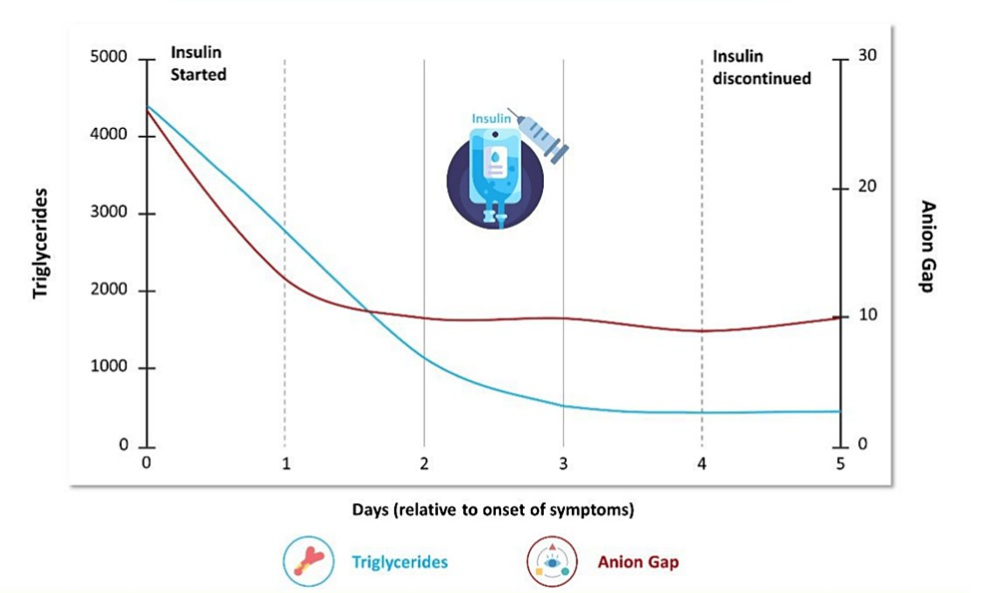
Insulin Response Over Time of Anion Gap and Triglyceride Levels

We admitted the patient to the intensive care unit and began insulin at 0.1 units/kg/hour for DKA resolution and HTG, with an impressive response (Figure 2). Plasmapheresis for severe HTG-induced pancreatitis was deferred. Continuous intravenous normal saline infusion was started at 250 ml per hour for both the treatment of DKA and pancreatitis. Hydromorphone was used for pain control. Initially, the patient was started on a nothing-by-mouth (NPO) diet and upgraded when triglyceride levels were below 1000 mg/dL. In the setting of superior mesenteric vein thrombus, anticoagulation is recommended; however, given the potential for hemorrhagic transformation of severe pancreatitis, anticoagulation with heparin was deferred.

After the closure of the anion gap, resolution of DKA, and normalization of triglyceride levels, the diet was advanced and the patient was subsequently discharged. He was discharged on metformin, glargine, fenofibrate, and rosuvastatin for long-term management of diabetes, hyperlipidemia, and hypertriglyceridemia.

Our patient was scheduled for follow-up at our primary care clinic 5-7 days after discharge; however, he presented two months later. Laboratory work on follow-up was pertinent for triglyceride levels of 1093 mg/dL and LDL levels that could not be calculated due to HTG. He has been compliant with fenofibrate 145 mg daily, and rosuvastatin 40 mg daily. We have added omega-3 fatty acids for better control of triglyceride levels. Workup for type 1 diabetes was ruled out after testing for antibodies. Diabetes is being well managed with metformin 500 mg twice a day and glargine 15 units at night.

## Discussion

Traditionally, clinical presentation and laboratory findings suffice for the diagnosis of pancreatitis, and a CT scan of the abdomen is not necessary. In scenarios where DKA is present, which typically presents with the abdominal pain seen in pancreatitis, a CT scan becomes important as the clinical presentation becomes unreliable. Imaging could also be helpful for identifying potential complications of pancreatitis, such as necrotizing or hemorrhagic transformation and thrombosis of the SMV. What otherwise would be attributable to symptoms and clinical appearance of DKA, may actually be complications of severe pancreatitis that would go undiagnosed without proper imaging. This approach should not be misconstrued as using CT for diagnosis; rather, it is to rule out or confirm serious complications that could significantly alter patient management. In 2021, a case report urged the need for imaging for proper diagnosis of pancreatitis in the setting of superimposed DKA [6].

Our case also advocates for the effectiveness of insulin therapy in the treatment of severe hypertriglyceridemia and potentially considering insulin as a preferable first-line treatment over plasmapheresis as an established guideline, especially when in the presence of overlapping DKA. A case report in 2023 urged the need for insulin infusion in the treatment of this unique circumstance as well [7]. Lastly, the management of SMV thrombosis with anticoagulation in this context is questionable. Typically, the presence of an SMV thrombosis warrants anticoagulation in those with hepatic decompensation or bowel ischemia [8]. However, our patient had neither so anticoagulation was judiciously deferred in our patient due to the heightened risk of progression to hemorrhagic pancreatitis. SMV thrombosis is shown to not be a poor prognosis of acute pancreatitis and is safely reversible with resolution of acute pancreatitis [9]. Thus, this is why we focused our treatment on the underlying pancreatitis despite the theoretical possibility of thromboembolism or bowel ischemia. This case brings together the management of HTG-induced pancreatitis with complications and concurrent DKA, necessitating a tailored approach to both diagnosis and treatment. 

## Conclusions

This case emphasizes the need for heightened awareness in identifying overlapping clinical presentations, such as gastrointestinal symptoms in DKA and pancreatitis. It highlights the importance of comprehensive CT imaging for accurate diagnosis and identification of serious complications like necrotizing pancreatitis and vein thrombosis in such complex scenarios. The effective use of insulin in rapidly lowering triglyceride levels advocates for its potential as a preferred initial treatment over plasmapheresis. Additionally, the careful balancing of anticoagulation therapy risks, especially in the presence of SMV thrombosis and potential hemorrhagic transformation in pancreatitis, is crucial in ensuring optimal patient outcomes.
